# Patient Accessibility and Budget Impact of Orphan Drugs in South Korea: Long-Term and Real-World Data Analysis (2007–2019)

**DOI:** 10.3390/ijerph17092991

**Published:** 2020-04-26

**Authors:** Se Hee Lee, Seung-Lai Yoo, Joon Seok Bang, Jong Hyuk Lee

**Affiliations:** 1 College of Pharmacy, Sookmyung Women’s University, Seoul 04310, Korea; 2 Department of Insurance Benefits, National Health Insurance, Wonju 26464, Korea; 3 Department of Pharmaceutical Engineering, Hoseo University, Asan 32499, Korea

**Keywords:** Orphan drug, rare disease, pharmaceutical expenditure, reimbursement, budget impact, budget controls, patient accessibility, health services accessibility

## Abstract

This study aimed to identify orphan drug accessibility and impact on pharmaceutical budgets in South Korea by analyzing the status of orphan drug designation, approval, reimbursement, and pharmaceutical expenditure. We analyzed the dataset on orphan drugs designated, approved, and reimbursed from 2007 to 2019 based on long-term real-world data. The designated and approved orphan drugs were 165 and 156, respectively, and 88 out of 156 approved products were reimbursed. Total expenditure on orphan drugs increased annually to account for about 1.44% of total pharmaceutical expenditure in 2018. Orphan drug expenditure per patient increased on average by 8.7% per year. The average annual cost of orphan drugs was USD 27,000–USD 47,000, with the maximum value of USD 260,000–USD 560,000. As there are a number of orphan drugs that have not yet been reimbursable after approval, a reimbursement policy should be established that considers the characteristics of orphan drugs. Since the rapid increase in orphan drug expenditure can be a potential threat to the insurance budget, budget management should also be considered. In conclusion, it is necessary to take preemptive measures to manage the health insurance budget efficiently while improving patient accessibility to orphan drugs.

## 1. Introduction

With improvements in medical and diagnostic technology, approximately 6000 to 8000 rare diseases have been identified, and the number of patients diagnosed with rare diseases has rapidly increased [1,2]. In addition, unmet needs for therapeutics are increasing as most rare diseases shorten the patient’s life expectancy by damaging the body and reducing the quality of life (QOL) [2,3]. Since the Orphan Drug Act was introduced in the United States (US) in 1983, most developed countries, including countries in the European Union (EU) and Japan, have enacted orphan drug (OD) legislation, which includes orphan drug designation, marketing approval, post-marketing monitoring, and financial incentives [1,4,5,6]. Moreover, the identification of many rare diseases and development of biotechnology promote research and development (R&D) and marketing approval of ODs [2,4,7,8]. Most rare diseases are characterized by high severity, low prevalence, and no alternative treatments, making it difficult to prove the safety and efficacy of ODs as it requires a longer time and higher R&D cost for clinical trials [9]. Due to high price and the small number of patients, pharmaceutical expenditure per patient is very high, and the total budget impact of individual ODs is relatively low [5,6,10,11]. However, as the number of OD products increases and their impact is gradually increasing, policy makers are facing difficulties in decisions concerning reimbursement and pricing [1,5,6,10]. Due to the high price and uncertainty of clinical outcomes, in countries where a health technology assessment (HTA) system is applied, patients may not be treated with ODs at a suitable time because reimbursement is not available without proof of cost-effectiveness [2,7]. This means that even if marketing approval is successful by making a lot of effort, failure of reimbursement can result in social losses, as patients lose treatment opportunities and pharmaceutical companies fail to retrieve R&D costs. Therefore, stakeholders, including policy decision makers, should continuously monitor the impact of ODs on the overall budget to manage patient accessibility and financial risk related to ODs appropriately.

The situation of OD-related legislation and reimbursement in South Korea is similar to that of other countries. In South Korea, financial incentives are provided for R&D activation of OD, and various benefits are provided in terms of marketing authorization and reimbursement to improve patient accessibility [12]. However, the South Korean government is also struggling with the budget impact from the gradually increasing high-priced ODs. In 2007, South Korea introduced the HTA system to thoroughly manage pharmaceutical expenditure while remaining sensitive to the budget impact of high-priced ODs [13,14,15].

Although many studies related to OD availability and budget impact have been released in countries such as the United States and EU countries, studies based on long-term and real-world data have not been conducted in South Korea. This study aimed to identify patient accessibility and pharmaceutical budget impact of ODs in South Korea by analyzing the status of OD designation, approval, reimbursement, and pharmaceutical expenditure from 2007 (the year HTA was implemented) to 2019.

## 2. Methods

The study was conducted on medicinal products designated, approved, and reimbursed as ODs in South Korea from 2007 to 2019. Since the data before 2007 were inaccessible, the datasets used in this study included the data from 2007, with the introduction of the HTA system in South Korea, to 2019.

### 2.1. Patient Accessibility of ODs

The study analyzed OD designation, approval, and reimbursement from 2007 to 2019, and calculated the ratio of the number of reimbursed products as of 1 April 2020 to approved products for each year. The data on the designation and approval status of ODs were retrieved from the website of Korea Ministry of Food and Drug Safety (KMFDS) [16]. Reimbursement status of approved ODs was identified on the website of Health Insurance Review and Assessment Service, South Korea (HIRA) [17].

### 2.2. Budget Impact of ODs

The study analyzed the budget impact of 88 OD products that were approved and reimbursed between 2007 and 2018. The reimbursement for the listed drugs started in 2009 and the budget impact in 2009 was insignificant. Thus, the period from 2007 to 2009 was excluded from the analysis. From 2010 to 2018, the study analyzed total pharmaceutical expenditure and OD expenditure by year, annual growth rate in expenditure, and the percentage of OD expenditure to total pharmaceutical expenditure. In addition, the annual cost of patients treated with ODs (annual per patient costs) and annual expenditure of individual ODs were analyzed. For the cost analysis, the dataset was collected using publicly available materials from sources such as websites (HIRA and National Health Insurance Service (NHIS), South Korea) and claims data from NHIS.

## 3. Results

### 3.1. Current Status of Patient Accessibility of ODs in South Korea

As of 31 December 2019, 290 ingredients were designated for orphan drugs in South Korea. From 2007 to 2019, designated ODs and approved ODs were 165 and 156, respectively, and 88 out of 156 approved products (56.4%) were reimbursed (Table 1). Of the 165 designated products, there may have been products that had not applied for a New Drug Application (NDA), and some products that were designated before 2007 may have been approved afterwards. In addition, some of the 156 approved products may have not applied for reimbursement. Since the lead time until reimbursement from approval might be about 1 to 3 years, the rate of reimbursement has dropped to 45% over the past 5 years (2015–2019). 

### 3.2. Total Pharmaceutical and Orphan Drug Expenditure 

**Figure 1:**
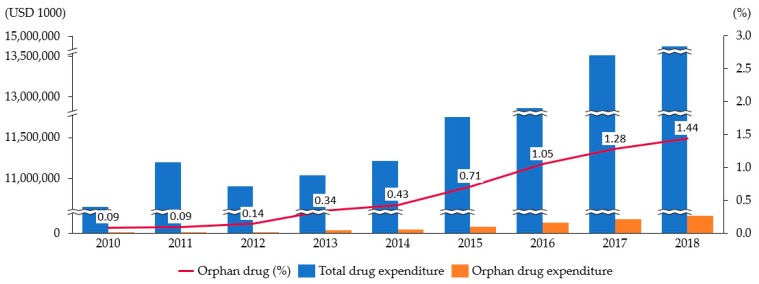
Trend of orphan drug (OD) expenditure ratio out of total pharma expenditure (2010–2018). The expenditure ratio on ODs gradually increased and accounted for about 1.44% out of total pharmaceutical expenditure in 2018. Source: Korea National Health Insurance Service, Health Insurance Claims Data.

As a result of analyzing the total annual pharmaceutical expenditure and the total number of ODs (88) reimbursed during the period 2010 to 2018, the expenditure on ODs gradually increased to USD 213,553,000 in 2018, which accounted for about 1.44% out of total pharmaceutical expenditure (USD 14,872,013,000; Figure 1). On the other hand, the compound annual growth rate (CAGR) of ODs was 47.8%, which was around 11 times higher compared to the growth rate of total pharmaceutical expenditure of 4.3% during the same period (Table 2).

### 3.3. Number of Patients Treated with ODs and Expenditure Per Patient of OD Products

The number of patients treated with ODs significantly increased each year, reaching 62,413 in 2018, with an average annual increase of 36.0%. The annual OD expenditure per patient annually increased to USD 4041 in 2017, but decreased to USD 3422 in 2018, showing an average increase of 8.7%. The main reason for a decrease in OD expenditure per patient in 2018 was that the patient number growth was mainly driven by products with lower cost and the average price of the newly listed products in 2018 was relatively lower than one of previous years. As a result of analyzing annual treatment costs of 88 ODs products, the average was USD 27,000–USD 47,000, with the maximum value of USD 260,000–USD 560,000 (Table 3).

## 4. Discussion

The most difficult policy hurdles on availability and accessibility of ODs relate to registration and reimbursement issues [1,2,7,18,19]. As the number of patients with rare diseases has increased, patient access to ODs has emerged as an important issue, increasing the number of designated and approved products. However, due to the high price of OD, there are still many OD products that are not included in the reimbursement after marketing authorization, which has a negative impact on patient accessibility [5,6,18,20]. In particular, countries that implement public health insurance, such as South Korea, may have an unfavorable view on spending a huge amount of money on a small number of patients. In other words, stakeholders related to reimbursement are struggling to adequately accept the conflict issues on efficient allocation of limited resources along with social obligations and ethical issues on patients in need of proper treatment [2,7,18,21,22,23]. As a result, some countries have introduced systems such as exemption of cost-effectiveness assessment, conditional reimbursement, flexible level of Incrementally Cost-Effectiveness Ratio threshold (ICER), and Risk Sharing Agreement (RSA) in HTA to enhance patient accessibility to ODs [1,17,24].

In South Korea, with the development of diagnostic technology, the number of patients with rare diseases has also been increasing every year: 2589 in 2002; 238,687 in 2008; 314,681 in 2010; and 520,970 in 2016 [12]. The Korean government enacted the Rare Disease Management Act in 2016 to comprehensively manage the tasks and treatments of patients with rare diseases [25]. In addition, OD-related regulations were introduced, such as tax favors for R&D, accelerated approval, exemption of data submission, and extension of market exclusivity period [12]. Moreover, various benefits for ODs are also provided in the HTA system. For instance, when the drug is designated as a medically essential drug, the drug can be reimbursed without proof of cost-effectiveness [26]. To increase OD accessibility, even if the drug, based on social needs and clinical values, is not designated as a medically essential drug, the value of the ICER threshold might be adjusted flexibly through a pharmaco-economic (PE) study assessment [26]. In addition, systems including RSA and PE study exemption are currently implemented to improve patient accessibility [26]. As a result of such legislation, OD availability and accessibility have improved significantly, but the accompanying budget impact has gradually increased as well. In South Korea, the proportion of pharmaceutical expenditure in national health insurance finance exceeds 20%, which is higher than the average pharmaceutical expenditure of 16% in Organization for Economic Cooperation and Development (OECD) countries, making stakeholders sensitive to increasing pharmaceutical costs [27].

In other words, the South Korean government is struggling to resolve OD accessibility and the efficient distribution of limited financial resources. Especially in South Korea, stakeholders may be sensitive to patient accessibility issues because of a number of high-profile cases (e.g., Novoseven RT, Presista, Myozyme, Elaprase, and Soliris). These ODs could either not make an agreement on price and budget limit in the final negotiation of reimbursement or had been refused supply by pharmaceutical companies due to low listing prices [28].

This study aimed to identify the progress of OD accessibility and budget impact in South Korea by analyzing the status of OD designation, approval, and reimbursement from 2007 (the year in which HTA was implemented) to 2019. Following growing trends in other countries, OD designation, approval, and reimbursement have been considerably improved in Korea under the influence of OD-related legislation. Since 2012, the number of designated, approved, and reimbursed products has increased, which can be explained by the effects of OD-related legislation applied to provide benefits for regulation and legislation of OD. However, the number of products is still less than that of other developed countries, such as the United States and countries in Europe, and many OD products are not approved after designation or are not reimbursable after marketing authorization [1,2,6,29]. 

As a result of analyzing the annual budget impact of 88 ODs approved and reimbursed during the same period, the number of patients treated with ODs continuously increased as expected, and the growth in OD expenditure was 11 times higher than total pharmaceutical expenditure. The reason for the excessive growth in total OD expenditure is due to the fact that the total amount is very small. As higher-priced ODs continue to be reimbursed, the number of patients and the amount of expenditure looks not reaching the steady state yet.

The OD expenditure rate of total pharmaceutical expenditure in South Korea is not as high as in the United States, Canada, and European countries, where the rate of expenditure reached 2.5%~8.9% in early 2010, although the methods and data sources are different [4,5,10,11,30,31].

On the other hand, the number of patients treated with ODs each year has increased with CAGR 36% (2010–2019), resulting in higher OD expenditure. The annual cost of individual OD products was up to USD 260,000–USD 560,000, which indicates that some specific products have very high annual expenditures and a very large proportion of the budget is spent on some patients treated with those OD. If only very few patients are treated with high-priced OD, this has a significant financial impact; if the number of patients increases for any reason, budget risk is inevitable, therefore the impact of ODs must be monitored continuously.

Although this study was based on long-term (2009–2018) real-world data on OD expenditure in South Korea, there are some limitations. Patient accessibility to ODs in South Korea should be compared with other countries to make it a more valuable study, but the scope was too wide to cover in this study. In addition, the datasets used in this study included data from 2007, the year of the introduction of the HTA system in South Korea, and thus the products designated before 2007 were not included. Therefore, the budget impact result in this study does not include all ODs that are currently being reimbursed. Moreover, for ODs with multiple indications, each orphan indication must be extracted and analyzed to obtain the exact budget impact of the OD, but currently there is no system in South Korea to extract budget data for each orphan indication. Despite these limitations, this study is the latest research on the availability, accessibility, and budget impact on ODs in South Korea, and can be used as an important reference for further research.

## 5. Conclusions

The South Korean government tried to improve the patient accessibility of ODs through various OD support policies, leading to an increase in the number of OD designations, approvals, and reimbursements. However, as there are a number of ODs that have not yet been approved or are not reimbursable after approval; a reimbursement policy should be established considering the disease characteristics of the ODs. Meanwhile, due to the increase in patients treated with ODs and high-priced OD products, the growth rate in OD expenditure was found to be very large, but the share in total pharmaceutical expenditure was not as large as that of major developed countries. However, as the rapid increase in OD expenditure can be a potential threat to the national insurance budget, budget management such as post-reimbursement monitoring of OD expenditure should be included. In other words, it is necessary to take preemptive measures to manage the health insurance budget efficiently while improving patient accessibility to OD.

## Figures and Tables

**Table 1:** Current designation, approval, and reimbursement status of orphan drugs in South Korea (2007–2019).

Year	2007	2008	2009	2010	2011	2012	2013	2014	2015	2016	2017	2018	2019	Total
Designation *	12	5	8	1	7	10	14	17	27	18	3	16	27	165
Approval **	8	14	5	14	15	15	10	15	23	14	8	6	9	156
Reimbursed ***	6	6	2	11	9	11	7	9	14	6	3	1	3	88
Reimbursed Ratio (%)	75.0	42.9	40.0	78.6	60.0	73.3	70.0	60.0	60.9	42.9	37.5	16.7	33.3	56.4

* As all designated products are not approved in the same year, designation year and approval year may differ. ** The number of approved products in a specific year may be greater than the number of products designated. *** Reimbursed is the number of reimbursed orphan drugs (ODs) as of 1 April 2020 among approved ODs in the year.

**Table 2:** Annual expenditure on pharmaceutical and orphan drugs (2010–2018).

Year	2010	2011	2012	2013	2014	2015	2016	2017	2018	CAGR (%)
Total pharma expenditure	10,641,174	11,190,798	10,895,323	11,034,395	11,207,612	11,748,808	12,857,228	13,508,153	14,872,013	4.3
OD expenditure *	9383	10,365	15,371	37,515	48,054	83,722	135,263	172,295	213,553	47.8
OD ratio (%)	0.09	0.09	0.14	0.34	0.43	0.71	1.05	1.28	1.44	NA

Source: Korea National Health Insurance Service, Health Insurance Claims Data; Expenditure unit: USD 1000. Exchange Rate: 1200 KRW/USD. CAGR: Compound annual growth rate, 2010–2018. * OD expenditure: Total expenditure of orphan drugs reimbursed since 2006.

**Table 3:** Annual number of patients treated with ODs and OD expenditure (2010–2018).

Year		2010	2011	2012	2013	2014	2015	2016	2017	2018	CAGR (%)
Patients no.		5348	6408	10,650	16,040	18,405	26,220	34,460	42,633	62,413	36.0
Annual total OD expenditure/patient		1754	1618	1443	2339	2611	3193	3925	4041	3422	8.7
Annual cost of each OD product	Mean	46,812	31,812	31,136	44,405	46,473	43,114	27,275	41,682	36,629	NA
	Min.	336	22	77	22	80	22	44	70	62	NA
	Max.	366,777	363,151	402,911	452,042	464,557	513,546	265,701	553,703	554,308	NA
	Median	3463	2187	2297	2076	2000	2161	2254	3808	4735	AN

Source: Korea National Health Insurance Service, Health Insurance Claims Data. Expenditure unit: USD; Exchange rate = 1200 KRW/USD. CAGR: Compound annual growth rate, 2010–2018.
